# Inhibition of induced-hepatic cancer *in vivo* through IQGAP1-shRNA gene therapy and modulation of TRAIL-induced apoptosis pathway

**DOI:** 10.3389/fonc.2022.998247

**Published:** 2022-10-05

**Authors:** Khairy M. A. Zoheir, Ahmed A. Abd-Rabou, Ahmed M. Darwish, Mohamed A. Abdelhafez, Karima F. Mahrous

**Affiliations:** ^1^ Cell Biology Department, Biotechnology Research Institute, National Research Centre, Cairo, Egypt; ^2^ Hormones Department, Medical Research and Clinical Studies Institute, National Research Centre, Cairo, Egypt

**Keywords:** IQGAP1, shRNA, liver cancer, *in vivo*, mouse

## Abstract

**Background:**

Liver cancer is the deadliest malignancy among common tumors. It is the top cause of cancer-related deaths in Egypt, and it is characterized by increasing occurrence among the population. The objective of this study was to determine the outcome of pre-treatment of IQGAP1-shRNA on induced mouse hepatocellular carcinoma model and evaluate the potency of this IQGAP1-shRNA plasmid to recover hepatic cancer as a new tool of cancer therapy. Therefore, we will use RNA interference (RNAi) technology to silence IQGAP1 oncogene to completely recover the chemically induced models for hepatic cancer by designing short RNAi specific for IQGAP1 gene in HCC cells *in vivo* and construct new vectors suitable for this purpose. We assigned mice into three groups: the first negative control group (NC) was injected with saline, the second control group was injected with shRNA (shNC), the third positive control group was injected with diethylnitrosamine (DENAA), and the fourth group was treated with the IQGAP1-shRNA prior to its exposure to DENA.

**Results:**

Our results revealed that the treated group with IQGAP1-shRNA with DENA developed very few cases of hepatic cancer when compared with the positive control group. The positive control group exhibited significant increases in the liver function level as well as a decrease in serum albumin levels when compared to both the treated and the negative control groups. The altered levels of the serum α-fetoprotein as well as of the tumor necrosis factor-alpha, and interleukin-4 in DENA-treated mice were significantly ameliorated by IQGAP1-shRNA administration. Flow cytometer analyses have indicated that the silencing of IQGAP1 cannot significantly modulate DENA-induced apoptosis in the circulating blood cells. Moreover, the elevated mRNA expression levels of IQGAP1, IQGAP3, KRas, HRas, interleukin-8, nuclear factor kappa B, caspase-3, caspase-9 and Bcl-2, were significantly decreased by the IQGAP1-shRNA treatment. However, the IQGAP2, DR4, DR5, p53 and BAX genes were found to be significantly up-regulated post-therapy. In agreement with these findings, IQGAP1-shRNA was able to modulate the DENA-induced histological changes in the mice liver which were represented by severe necrosis and hydropic degenerative changes.

**Conclusion:**

Our study revealed that IQGAP1-shRNA was able to preserve hepatocyte integrity and the liver histological architecture through the regulation of the expression of IQGAPs, Ras, TRAILs and IL-8 receptors, as well as of pro-apoptotic and anti-apoptotic genes. Therefore, the silencing of IQGAP1 could be part of a promising therapeutic strategy against hepatic cancer.

## Introduction

Patients with hepatic cancer develop resistance to conservative chemotherapeutic treatments quickly, and surgery signifies the only potentially therapeutic treatment. Hepatocarcinoma (HCC) is one of the most important health-threatening problems globally, as approximately more than fifty million cases are recognized annually.

The IQ motif-containing GTPase-activating protein 1 (IQGAP1) is a 189 kDa highly-conserved scaffold protein engaged in numerous cellular activities (including cell migration, cell invasion, cell adhesion, and cell proliferation) (1). As one of its utmost presumed partners, small GTPases team up with IQGPA1 to control the cytoskeletal reorganization and cell connection in cancer cells that enhance cancer development. Thus, the variation of the mechanistic ways that connect IQGAP1 with small GTPases could furnish cancer therapies on new foundations (2).

The IQGAP family has two other proteins (namely, IQGAP2 and IQGAP3); of these, IQGAP2 appears to possess tumor-suppressive properties. Indeed, IQGAP1 has been reported to rise at both the mRNA and protein levels in an assortment of cancers, and its levels have been associated with cancer aggressiveness (3). However, the role of IQGAP1 in tumor development and metastasis remains unclarified (4).

Notably, the IQGAP1 knockdown prohibits the invasion of human ovarian carcinoma HO-8910PM cells in vitro (5), and of human breast cancerous MCF-7 cells from in vitro to in vivo applications (6). The knockout of IQGAP1 from cells has been shown to make B-Raf cells insensitive to the epidermal growth factor stimulation, while a B-Raf knockdown cited that of IQGAP1 has been shown to exert higher kinase activity when compared to free B-Raf (7). Undoubtedly, a siRNA-induced IQGAP1 knockdown can significantly diminish the vascular endothelial-derived growth factor (VEGF)-stimulated angiogenesis in vivo (8). Hebert et al. have reported that a drop in IQGAP1 levels can shorten the formation of metastases without limiting primary or metastatic tumor growth in IQGAP1-knockdown and -knockout experiments conducted on human melanoma and breast cancer cells (4). In addition, they were able to establish that IQGAP1-knockout cells are significantly lacking in extravasation capacity. These data firmly establish IQGAP1 as a critical metastasis promoter acting through the regulation of extravasation in vivo.

It has been pointed out that differentially expressed genes emerge because of an IQGAP1 down-regulation along with enrichment in chemotaxis and cytokine signaling, as well as a reduction in the immune response (4).

Mechanistic analysis has DENAoted that the Cdc42/Rac1 pathway might be part of the IQGAP1-mediated-pancreatic cell proliferation and tumorigenesis (9). Furthermore, Liu et al. (9) and Zhang et al. (10) have recently remarked that the E3 ubiquitin ligase mind bomb 1 (MIB1) can promote pancreatic cancer progression through the suppression of tumorigenicity 7 (ST7) degradation, followed by a down-regulation of IQGAP1 in pancreatic cancer cells (8).

The knockdown of IQGAP1 is known to block both Rac1 and Cdc42, thus mediating migration and invasion of glioma cells, and consequently putting down various other components of the invasion process (namely, matrix metalloproteinases) (11, 12). Moreover, IQGAP1 expression seems to be suppressed by the TGF-β signaling and myofibroblasts accompanying human colorectal liver metastases that are characterized by a downregulated expression of IQGAP1 (13).

Together, IQGAP1 and the heterogeneous nuclear ribonucleoprotein M (hnRNPM) have been shown to stimulate gastric cancer cell growth, while their genetic depletion prompts cell cycle arrest that subsequently causes tumor progression (14). IQGAP1 mRNA levels have been identified as being upregulated in many malignant cell types (15). The IQGAP1-knockdown can markedly repress colorectal cancer (CRC) cell migration and invasion in vitro (16). Otherwise, the cell growth, cell migration, and tumorigenesis of CRC have been seen to be fostered by the extracellular signal-regulated kinase pathway, which is activated by a SUMOylated IQGAP1 (17). This is in agreement with the observed overexpression of IQGAP1 associated with the invasiveness of CRC, and especially in advanced carcinomas in which the invasive capability of cancer cells can be detected (18). Mo et al. have suggested that IQGAP1 is a predictive indicator and a new therapeutic target for HCC patients (19).

Furthermore, Lin et al. have provided us with the first proof for a significant association of IQGAP1 with clear cell renal cell carcinoma mortality. In fact, IQGAP1 down-regulation has been shown to maintain links with network shifts consisting of 611 differentially expressed genes (20).

Similarly, Wei et al. (21) have investigated the role of IQGAP1 in head and neck cancer (HNC), by matrix approaches on different models. They have indicated that IQGAP1 is vital for the effective PI3K signal and that the IQGAP1 loss can significantly diminish cancer progression in vivo. The PI3K signaling that is originally downregulated in HNC can be seen as upregulated in IQGAP1-knockout mice, suggesting that IQGAP1 takes part in HNC. More importantly, high IQGAP1 levels have been associated with deprived survival in HNC patients. As a result, drugs that target IQGAP1 are believed to be able to generate new approaches for the treatment of HNC (22). Overall, although IQGAP1 expression is not essential for HCC growth, the gain of IQGAP1 function helps the rapid onset and increased liver carcinogenesis (23). Other results show that an adequate amount of IQGAP1 scaffold is necessary to maintain the quiescent status of the liver. Nonetheless, to the best of our information, there are no studies focusing on the IQGAP1-knockdown involvement in the inhibition of hepatic cancer in mice. This study aimed to uncover new avenues for the treatment of hepatic cancer *in vivo* through the silencing of IQGAP1 and the assessment of its effects on critical inflammatory as well as pre- and antiapoptotic signaling parameters.

## Methods

Authors confirm that all methods were carried out in accordance with relevant guidelines and regulations. Authors confirm that all methods are reported in accordance with arrive guidelines. The animals were kept following the National Institute of Health (NIH) Guide for the Care and Habit of Laboratory Animals. The Medical Research Ethics Committee has approved this study (Project number: 12060168).

### Animals

A hundred 21-day-old male Balb/c mice were purchased from the Animal Care Center and were kept in polycarbonate cages, in an animal facility that is accredited by the national authority for laboratory animal care evaluation and accreditation. All mice were fed with an NIH-07 diet (roDENAt chow) and water *ad libitum*. Before the undertaking of any tests, the animals were allowed to acclimatize for 7 days.

### Chemicals

The IQGAP1-knockdown was performed according to the protocol reported in our previous study, with a few modifications based on Zoheir *et al.* (24). The IQGAP1 sequence (NC_000015.10) was obtained from the NCBI gene bank.

Diethylnitrosamine (DENA) was purchased from Sigma Chemicals (St. Louis, MO, USA). The TRIzol reagent was purchased from Invitrogen (Carlsbad, CA, USA), while the high-capacity cDNA reverse transcription kit, the primers for real-time PCR, and the SYBR Green Universal Master mix were all bought from Applied Biosystems^®^ (Foster City, CA, USA). Antibodies specific for b-actin, IQGAP1, and p53 were purchased from Santa Cruz Biotechnology.

### Construction of shRNA-I QGAP s-P- vectors

IQGAP -targeted siRNAs vector was constructed using the RNAi-DNA vector technique according to Promega Ribomax Large Scale RNA Production System T7.

The oligonucleotides of the short hairpin RNA (shRNA) were annealed and inserted into the BamHI and EcoRI sites of the RNAi-Ready PSIREN-Retroozs Green Vector (BD Biosciences, Clontech, CA). This plasmid encoding variant 1 of the mouse/mouse IQGAP1 was obtained from Santa Cruz Company (IQGAP1 siRNA (m) sc-35701 SH).

The empty vector pTZU6 _ 1 was used as a negative control. Other group of mice was treated by P- 1, QGAP –shRNA before induction hepatic cancer with DENAA IQGAP1 -shRNA, or p-shRNA (50ng) with LipofectamineTM2000 was directly injected into the tumor *in situ*.

### Experimental plan

After acclimation, the 21-day-old male Balb/c mice were randomly divided into four experimental groups. The first experimental group of mice was injected only with saline (negative control NC group), the second control group was injected with shRNA (shNC), the third experimental group of mice was injected intraperitoneally (i.p.) with DENA (that was dissolved in saline) at a dose of 50 mg/kg on the experimental days 0, 7, 14 and 21 (positive control group), and the fourth experimental group of mice was injected with the IQGAP1-shRNA vector and a day later they were admitted to the same DENA-administrating protocol as the previous positive control group (treated group). Mice were euthanized by CO_2_ asphyxiation and exsanguination and were subsequently weighed and necropsied on experimental day 288 (after the injection of DENA). Every 4 weeks, the occurrence of cancer was examined by a measurement of the serum α-fetoprotein (AFP; a tumor marker) and histopathology by choosing random mice from each group. In addition to blood, the mice’ livers, lungs, and hearts were removed, weighed, and examined to identify the existence of any grossly visible lesion. Moreover, these livers were dissected to separate their lobes and were subsequently cut into 1- to 2-mm slices. A piece from each lobe was then fixed in formalin for 48–72 h and then immersed in paraffin resulting in a total of three paraffin blocks for each animal. Serial sections from each block were later stained with hematoxylin and eosin (H&E). Hepatic preneoplastic and neoplastic lesions were categorized based on the mouse pathology.

### Histopathological examination

The mouse liver morphology was examined by histopathology. Liver sections (of 5-micron thickness) were fixed and stained with regular H&E stain, and then inspected under a light microscope, and lesions were photographed with an Olympus digital camera that was installed on the Olympus microscope with a 1/2 X photo-adaptor of a 100x objective lens.

### Quantitative analysis of altered hepatocellular foci

Quantitative analysis of the AHF was achieved by operating a two-dimensional evaluation method. The whole H&E staining sections were scanned by ScanScope CS (Aperio Technologies, Vista, CA, USA). Photos were then observed through Aperio’s ImageScope viewer software (Aperio Technologies), on which the quantitative analysis was performed. The number and the volume of each focus (eosinophilic, clear, and basophilic), as well as the total volume of the examined liver sections, were quantified. The multiplicity of AHF was then stated as numbers per centimeter squared, and the volume of the foci was represented as the percentage of total liver volume (%).

### RNA extraction and cDNA synthesis

Under the manufacturer’s guidelines, the total RNA from the hepatic tissue homogenate was isolated by using the TRIzol reagent (Gibco, Invitrogen; Eugene, OR, USA) according to Zoheir et al. (24),, Along with the manufacturer’s instructions of applied Biosystems kit, the first-strand cDNA synthesis was operated utilizing the high-capacity cDNA reverse transcription kit (Applied Biosystems^®^; Foster City, CA, USA).

### Quantification of mRNA expression *via* real-time polymerase chain reaction

According to (25) the quantitative analyses of target genes’ mRNA expression were undertaken by employing RT-PCR. Briefly, the resultant cDNA from the above preparation was exposed to PCR amplification within 96-well optical reaction plates in the ABI Prism 7500 System (Applied Biosystems). The employed primers in this study were selected from the website pubmed.com (http://www.ncbi.nlm.nih.gov/tools/primer-blast) as listed in **Table 1**. The RT-PCR data were analyzed by using the relative gene expression (i.e., ΔΔCT or 2^-ΔΔCT^) method, as illustrated in Applied Biosystems User Bulletin No. 2. With respect to the ΔΔCT of the 2^- ΔΔCT^ method, the first ΔCT is the difference in threshold cycle between the target and reference genes. ΔCT = CT(a target gene)−CT(a reference gene). ΔΔCT = ΔCT (a target sample)−ΔCT(a reference sample) The data are herein presented as the fold change in gene expression, as normalized to the endogenous reference gene (β-Actin gene) and relatively to a calibrator.

**Table 1:** Plasmid and primer sequences of mouse genes.

Sequence (reverse)	Sequence (forward)	Name
Anti-sense UUCAGCAAGAUCACUGUGGtt UUCAGCAAGAUCACUGUGGtt UACAACUUCAUCUUUGUGGtt	sc-35701A: Sense sc-35701A: CAGUGAUCUUGCUGAAtt sc-35701B: CAGUGAUCUUGCUGAAtt sc 5701B:CCACAAAGAUGAAGUUGUAtt	IQGAP1 shRNA Plasmid (m) (sc-35701 SH)
R: AGCCCATAGTGGAGTGGGAT	F: CTAGGCATCTTCGTCCGTCC	IL-8
R: CCGTACCAGAGCGAGATGAC	F: GGGGAGCTTGGAACGCTAAG	Casp3
R: ACGATCACAAGGAGGAAG	F: GCTGAAGAGACAATGAAC	DCR1
R: CCTTTCCCCTTCCCCCATTC	F: CTGGATCCAAGACCAGGGTG	Bax
R: GCCATCCAAGGTCTCGATGT	F: AGAACGACCTGACTGCCAAG	Casp9
R: GCCACACGTTTCTTGGCAAT	F: AGCATGCGACCTCTGTTTGA	Bcl-2
R: TCGTCAGCTGAGTCGTTTCC	F: CACTGACGGGGAAGAGGAA	DR5
R: GCATCGTCAACACCCTGTCT	F: AGACACGAAACAGGCTCAGG	Kras
R: TCCATGCGAAGGTCTTGGTC	F: CTGACACCAGGCTCAGGACA	Hras
R: TTCGCGTCCGGCTTCCTCAAG	F: ATGAACTCACTGGTTTCTTGGC	DR4
R: GGGTCAAGTACTGGACTG	F: AGCTGTGGTTGTGGTTGG	DCR2
R: GCTCTTCGCTAACGGCATT	F: GCCCTGCTCCCTCATTTTCT	IQGAP1
R: CTGCAAGGTGACCAGAGTGT	R: TGTCACCATTCGGAACCAGG	IQGAP3
R: TAGTGCAGCTTCGTCCACAG	F: TAGTGCAGCTTCGTCCACAG	IQGAP2

### Western blot analysis

To confirm the gene expression data, we carried out Western blotting for the analysis of the presence of two proteins (IQGAP1 and interleukin-8; IL-8) in the liver tissue. The total proteins were extracted from liver tissue by a method as described by (24). Protein concentrations were measured by the Lowry method (26). IQGAP1 and IL-8 antibodies (catalog number: sc-376021 and sc-376750) as well as those against β-actin (catalog number: sc-47778) were diluted in PBST containing 5% non-fat milk and were incubated overnight with the membranes at 4°C. Primary antibodies of IQGAP1 and interleukin-8 were purchased from Invitrogen (Carlsbad, CA, USA. Primary antibodies were diluted in TBST according to manufactured instructions. Incubation was done overnight in each primary antibody solution, against the blotted target protein, at 4°C. The blot was rinsed 3–5 times for 5 min with TBST. Incubation was done in the HRP-conjugated secondary antibody (Goat anti-rabbit IgG- HRP-1mg Goat mab -Novus Biologicals) solution against the blotted target protein for 1 hr at room temperature. The chemiluminescent substrate (ClarityTM Western ECL substrate Bio-Rad cat#170-5060) was applied to the blot according to the manufacturer’s recommendation. The chemiluminescent signals were captured using a CCD camera-based imager. Image analysis software was used to read the band intensity of the target proteins against control sample beta actin (housekeeping protein) by protein normalization on the ChemiDoc MP imager. All fold changes of band densities were determined with normalization to β-actin, an endogenous control. Relative protein expression was calculated as relative density of a protein band normalized to the endogenous control. Each experiment was conducted in triplicate and repeated three times independently. The blots were visualized using a chemiluminescence detection kit (ECL; Millipore), and the densitometry scanning quantified the optical density of each band.

### Cytokine secretion analysis

Supernatants obtained from the mice sera were cleaned by centrifugation (2000 g, 10 min), and cytokine levels were measured using commercially available ELISA kits: TNF-α and IL-4. Cytokine levels in the serum were estimated by the LEGENDplexTM multi-analyte flow assay kits (BioLegend, San Diego, CA), following the manufacturer’s instructions. Data were gathered on the MACSQuant^®^ Analyzer 10 (Miltenyi Biotech, Germany) and were subsequently analyzed by the LEGENDplex Data Analysis V8 software.

### Biochemical analyses

The activities of alanine aminotransferase (ALT) and alkaline phosphatase (ALP) in the serum were measured by a kinetic method with a kit of the BioSystem Company (Spain), as stated in the materials and methods of (27). Total bilirubin levels in the serum were determined by a colorimetric procedure by using kits of Human Gesellschaft für Biochemica und Diagnostica mbH, WiesbaDENA, Germany, as described in (27). The serum albumin level was estimated by a colorimetric method with the use of reagent kits purchased from the Diamond Diagnostics Company, according to (28). Serum AFP and serum tumor necrosis factor-alpha (TNF-α) levels were measured by a colorimetric method with kits developed by Quantikine following the manufacturer’s instructions. Serum IL-4 levels were assayed with a RayBio^®^ Mice IL-4 ELISA kit. The liver glutathione (GSH) content was determined according to (29). The glutathione-S-transferase (GST) activity in the liver was detected according to (30). The liver glutathione peroxidase (GPx) activity was pinpointed, and the liver lipid peroxidation (LPO) content was identified by measuring thiobarbituric acid reactive substances (TBARS) according to the method of (31). Finally, the liver superoxide dismutase (SOD) activity was assayed by following the technique of (32).

### Flow cytometer analysis

This assay was executed with Annexin V FITC and propidium iodide (PI) to measure the apoptosis of the circulating blood cells. The apoptotic analysis was dedicated to discriminating between early and late apoptotic cells, as well as necrotic cells. According to standard protocol (24) which was modified by (33), the apoptosis of the treated and the untreated cells was assessed through a flow cytometer instrument (BD Biosciences, USA). The gating of the required cell population was carried out according to the standard procedures of the flow cytometer.

### Statistical analysis

Data were expressed as mean ± standard error. One-way analysis of variance (one-way ANOVA) was used to identify statistically significant differences within groups. One-way-ANOVA followed by a Tukey’s test was used in order to perform comparisons among various groups at p < 0.05. All statistical analyses were done with the use of the SPSS 15.0 software. ^a^ indicated high significantly difference at P <0.01 between DENA-induced mouse and normal control. ^b^ indicated significantly difference at P <0.05 between DENA-induced mouse and normal control. ^c^ indicated significantly difference at P <0.05 between IQGAP1-shRNA treated group and DENA-induced mouse.

## Results

### Histological changes

**Figure 1:**
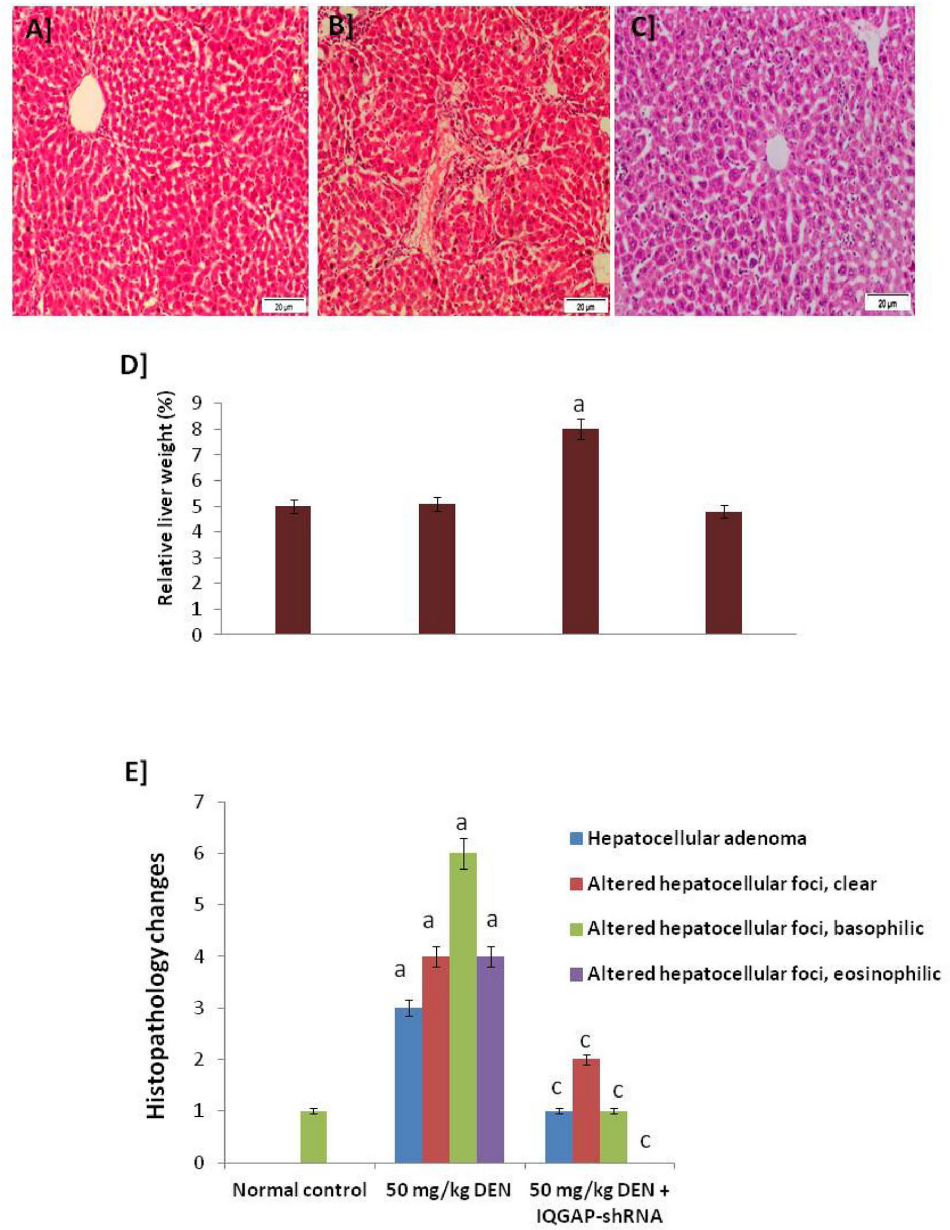
Histopathology changes. **(A)** A photomicrograph of a section of control liver showing the normal structure of the hepatic lobule. The central vein lies at the center of the lobule and intact with the hepatocytes with strong stained eosinophilic granulated cytoplasm, and distinct nuclei. In between the strands of hepatocytes, the hepatic sinusoids are shown **(B)** A micrograph of liver section from mice that given DENA only showing complete destruction of the sinusoidal architectural pattern. Hepatocellular carcinoma: a mononuclear inflammatory cell infiltrate extends from portal areas and disrupts the limiting plate of hepatocytes, which are undergoing apoptosis **(C)** A micrograph of a section of positive-control liver mice that given DENA and treated with IQGAP1-shRNAL plasmid showing improvements in the structure of liver that appeared more or less like normal one. Complete arrangement of the sinusoidal architectural patterns, no mononuclear inflammatory cells are shown (H & E stain). **(D)** Relative liver weight (%) to body weight. **(E)** Histopathology changes in normal, induced, and treated tissues.a indicated high significantly difference at P <0.05 between treated mouse and normal control.

Liver histological sections of the normal group (negative control group), the DENA-injected group (positive control group), and the DENA-injected group treated with IQGAP1-shRNA (treated group) are presented in **Figures 1A-C**.

The photomicrographs of the liver sections of the negative control group mice revealed a normal histological structure of the hepatic lobule. The liver of animals injected with DENA showed severe hydropic degenerative changes and fatty change of hepatocytes. Yet, the liver of animals injected with both DENA and IQGAP1-shRNA presented with a nearly-normal histological phenotype and Kupffer cell proliferation (**Figures 1A-C**).

**Figure 1D** shows the significant changes in relative liver weight of the mice in different tested groups, recording that the relative liver weight of the treated mice presented with a nearly-normal percent with high significant changes compared to DENA-induced model.

**Figure 1E** shows the significant changes in the histological features which confirmed that the liver of animals injected with both DENA and IQGAP1-shRNA presented with a nearly-normal histological phenotype and the Altered Hepatocellular Foci (AHF) returned back slightly close to the healthy control.

### Effects on serum liver function-related parameters

There were variations in several serum biochemical indicators linked to liver function. The injection of DENA caused a significant (p < 0.05) rise in total bilirubin levels and serum ALT and ALP activity. When mice were given IQGAP1-shRNA treatment and subjected to DENA, these levels fell. The mice who had received DENA responded better to the treatment with IQGAP1-shRNA in terms of lowering their high ALT activity and total bilirubin levels. However, the serum albumin level (**Table 2**) significantly decreased in the mice that received DENA. The latter group’s low serum albumin levels were considerably raised by IQGAP1-shRNA therapy.

**Table 2:** Liver function markers.

Treatment	ALT	ALP	Total bilirubin	Albumin
Normal control	40±2	45±3	1±0.1	40±3.5
50 mg/kg DEN	85±5.5^a^	90±6.5^a^	3.5±0.5^a^	15±2.5^a^
50 mg/kg DEN + IQGAP-shRNA	50±3.5^c^	48±4.5^c^	1.3±0.2^c^	33±3.0^c^

^a^indicated high significantly difference at P <0.01 between DEN-induced mouse and normal control. ^c^ indicated significantly difference at P <0.05 between IQGAP1-shRNA treated group and DEN-induced mouse (n=8).

### Changes in serum tumor markers

**Table 3** shows the alteration in serum AFP levels. The recorded percentage was found to be significantly increased in comparison to that of the treated group and negative control group, and serum AFP levels were found to be significantly (p < 0.05) higher in mice that had received DENA.

**Table 3:** Inflammatory and anti-oxidant candidates .

Treatment	TNF-α	IL-4	AFP	GST	SOD
Normal control	12±3.0	0.025± 0.001	15±3.2	5.2±1.05	0.82±0.05
50 mg/kg DEN	25±5.1^a^	0.5± 0.005^a^	75±4.5^a^	1.4±0.2 ^a^	0.09±0.004^a^
50 mg/kg DEN + IQGAP-shRNA	15±3.5^c^	0.05± 0.001^c^	20±2.8^c^	4.1±0.5 ^c^	0.78±0.06^c^

^a^indicated high significantly difference at P <0.01 between DEN-induced mouse and normal control. ^c^ indicated significantly difference at P <0.05 between IQGAP1-shRNA treated group and DEN-induced mouse (n=8).

### Effects on inflammation-related serum markers

The recorded changes in serum TNF-α and IL-4 levels are presented in **Table 3**. Serum TNF-α level were found to be significantly (p < 0.05) elevated in the DENA-administered mice, and IL-4 levels were found to be significantly (p < 0.05) increased because of the administration of DENA. Moreover, the treatment of DENA-poisoned mice with IQGAP1-shRNA resulted in a lowering of the elevated serum TNF-α level, and into amelioration of the higher serum IL-4 levels.

### Effects on liver oxidative stress and antioxidant defense biomarkers

The administration of DENA produced a significant decrease in GST level. The treatment of these animals with IQGAP1-shRNA yielded a significant (p < 0.05) upsurge in the GST content as compared to the DENA-treated mice group. The treatment of DENA-poisoned mice with IQGAP1-shRNA induced a significant increase in their liver GST activity (**Table 3**).

In **Table 3**, the liver SOD activity was found to be significantly decreased because of DENA administration. Nevertheless, the administration of IQGAP1-shRNA was able to induce a significant (p < 0.05) increase of the aforementioned lowered SOD activity. The treatment with IQGAP1-shRNA was even more effective in improving the lowered liver GSH content that was induced by DENA.

### Effects on hepatic mRNA expression of IQGAPs and Ras

**Figure 2:**
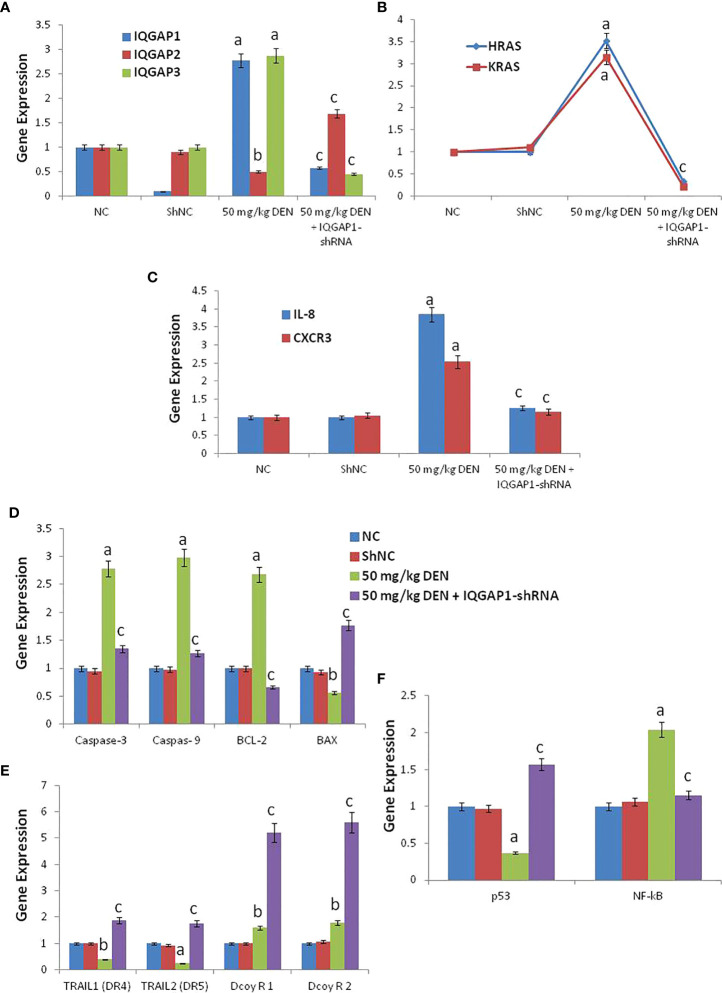
The RT-qPCR validation of mRNA expression for IQGAP1, IQGAP2, IQGAP3, KRas, HRas, IL-8, CXCR3, caspase-3, caspase-9, Bcl-2, BAX, DR4 (TRAIL1), DR5 (TRAIL2), DecoyR1, DecoyR2, p53, and NF-kB **(A-F)** in liver tissue among groups of control, DENA and IQGAP1-shRNA plasmid. NC; normal control and ShNC; IQGAP1-shRNA control. ^a^ indicated high significantly difference at P <0.01 between DENA-induced mouse and normal control. ^b^ indicated significantly difference at P <0.05 between DENA-induced mouse and normal control. ^c^ indicated significantly difference at P <0.05 between IQGAP1-shRNA treated group and DENA-induced mouse. Error bars represents standard error of mean (SEM). Means comparisons were performed by using One-Way ANOVA test.

The administration of DENA increased the mRNA levels of IQGAP1, IQGAP3, HRas, and KRas, whereas downregulated IQGAP2. However, the injection of the shRNA modulated these genes, through a decrease of the mRNA levels of IQGAP1, IQGAP3, HRas, and KRas, and an increase of the mRNA levels of IQGAP2, when compared to both negative control and shNC group (**Figures 2A, B**).

### Effects on hepatic mRNA expression of IL-8 and CXCR3

Mice treated with DENA exhibited increased hepatic IL-8 and CXCR3 mRNA levels, but the treatment with IQGAP1-shRNA lowered these increases to levels closer to those of the negative and shNC group (**Figure 2C**).

### Effects on hepatic mRNA expression of caspase-3, caspase-9, BAX, and BCL-2

The mRNA levels of caspase-3 and -9 and of BCL-2 were found to be highly increased in DENA-treated animals, but the mRNA levels of BAX were decreased. The animals that received the IQGAP1-shRNA exhibit this deregulation (**Figure 2D**).

### Effects on hepatic mRNA expression of TRAILs (DR4 and DR5) and decoys

The expression levels of DR4 and DR5 were found to be highly down-regulated in the positive DENA control group, while the administration of IQGAP1-shRNA upregulated the expression of these two genes as compared to the negative and shNC group (**Figure 2E**). In addition, we found that both Decoy R1 and Decoy R2 were highly up-regulated in both positive DENA control and IQGAP1-shRNA treated group if compared with negative and shNC control. Whatever, there was significant differences between DENA positive control and IQGAP1-shRNA treated group for both Decoy R1 and Decoy R2 as shown in (**Figures 2D-F**).

### Effects on hepatic mRNA expression of P53 and NF-K B

The expression of P53 was down regulated in DENA treated animals but NF-K B, was up regulated. The animals that received the IQGAP1-shRNA modulated these two genes, (**Figure 2F**).

### Effects on hepatic protein expression of IQGAP1 and IL-8

**Figure 3:**
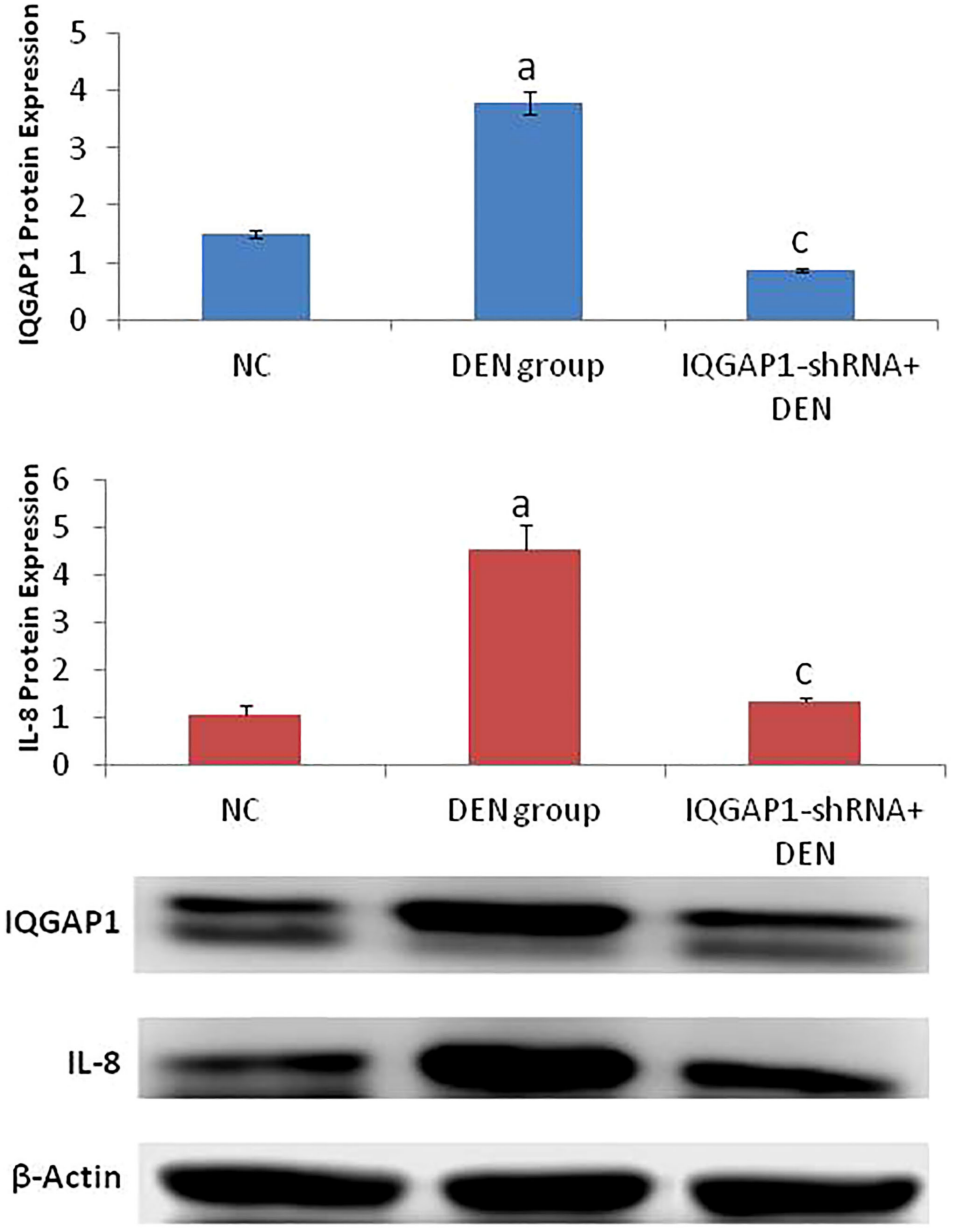
Protein expression of both IQGAP1 and IL-8 in liver tissues by western blot. ^a^ indicated high significantly difference at P <0.01 between DEN-induced mouse and normal control. ^c^ indicated significantly difference at P <0.05 between IQGAP1-shRNA treated group and DEN-induced mouse. Error bars represents standard error of mean (SEM). Means comparisons were performed by using One-Way ANOVA test.

Both IQGAP1 and IL-8 were highly expressed in the DENA-treated (positive control) group, but in the treated group, we were able to identify a potent decrease of the IQGAP1 protein levels only when compared to those of the negative control group (**Figure 3**).

### IQGAP1-knockdown and apoptosis in the circulating blood cells

**Figure 4:**
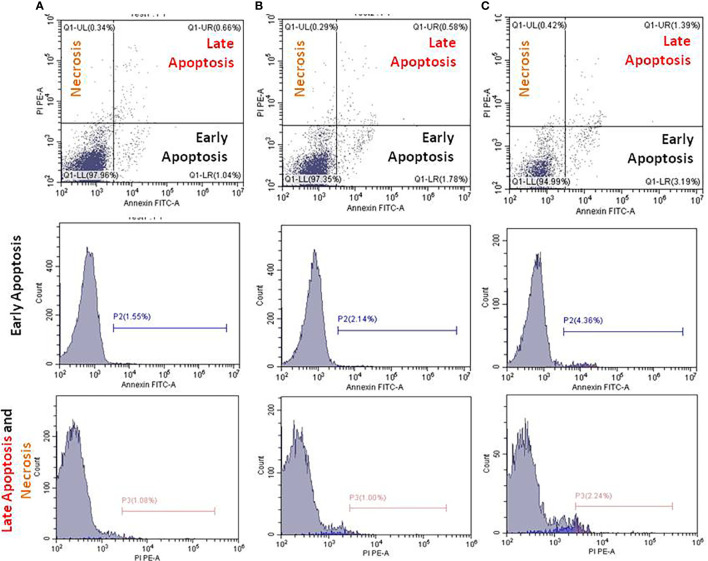
Flow cytometry analysis using Annexin V FITC and propidium iodide (PI) for apoptosis measurements. Apoptosis of the untreated **(A)**, DENA injected **(B)**, and IQGAP1-shRNA vector treated circulating cells in the blood stream were measured in mice **(C)**. Charts in each column represent cells underwent early, late apoptosis, and necrosis.

The underlying mechanisms of the herein attempted IQGAP1-knockdown on the circulating blood cells were determined by employing flow cytometer. In the present study, the early apoptotic, late apoptotic, and necrotic cells were annotated with Annexin V, Annexin V/PI, and PI, respectively. We identified an increased number of early and late apoptotic cells in the positive control group when compared with both the treated and negative control groups (**Figure 4**). In the DENA-treated group, late apoptosis was accompanied by an increase in necrotic cell numbers, and this was the case in the other two groups. The number of these cells was found to be greater than that of the early apoptotic circulating blood cells (*p* < 0.05).

## Discussion

Discover a new tool for the prevention of hepatic cancer *in vivo* through the silencing of IQGAP1 mRNA and the evaluation of its effects on serious inflammatory as well as pre- and antiapoptotic signaling parameters was our aim of this study.

Hepatic cancer is a common malignancy of the digestive system that affects human health (34). Liver cancer is one of the most common malignancies globally, and the patient survival rate remains poor. The control of liver disorders is challenging for modern medicine (35). In order to treat HCC, new surgical procedures are paired with radiotherapy and/or chemotherapy; nonetheless, the overall survival rate of HCC patients is not improved by using these treatments. Therefore, the discovery of novel approaches aiming to cure HCC with improved survival rates is necessary to address this issue. IQGAP1 acts as an oncogenic regulator. IQGAP1 is oncogenic and its up-regulation leads to the induction of liver cancer so the inhibition or knockdown of the expression of IQGAP1 is a significant reason for prevention of the initiation and development of hepatic cancer (5). Therefore, the mechanism regulating the knockdown of IQGAP1 could provide a new tool for the biological treatment of HCC. New advances have shown that IQGAPs exhibit an irregular expression in HCC tissues and that their deregulation contributes to the HCC initiation and progression. Researchers have suggested that IQGAP1 is oncogenic, and its upregulation leads to the induction of liver cancer, while IQGAP2 acts as a suppressor gene (36, 37).

For years, the medical community has called chronic or life-threatening diseases “hopeless,” but now, gene therapy offers hope to those looking for relief from hundreds of different diseases. The use of RNAi is one of the most promising cancer therapies and our way to wellness. As mRNA is employed in place of protein therapy nowadays, RNAs are used as medication to treat genetic disorders, autoimmune diseases, cancer, infectious diseases, and other ailments (37). In this study, we have methodically estimated the mechanisms and functions of the IQGAP1- shRNA in a mouse model of HCC and discussed the existing gaps and the future direction for studies on liver cancer-related IQGAP1-shRNA. Consequently, our study was planned to assess the potential preventive effects and the mechanism of action of IQGAP1-shRNA against DENA-induced HCC. Moreover, this application aimed at determining the outcome of inhibiting the IQGAP1 gene and, consequently, its effect upon Il-8 and its receptor family genes, caspases (3 and 9), BAX, Bcl-2, and TRAIL-induced apoptosis in the mouse liver cancer. The study also aimed to delineate the mechanism of such modulations (a result of oxidative stress, modulations in the antioxidant defense mechanisms, and/or apoptosis). IQGAP1 is an inducer gene for cancer, and it has been reported to induce liver cancer in both humans and animals. Several studies have reported that the connections of IQGAP1 with multiple cellular proteins have been shown to regulate several cellular functions (38). The upregulation of IQGAP1 has been recorded in a variety of human cancers, including female-specific tumors (gynecological malignancies) (39), pulmonary cancer (40), pancreatic cancer (41), hepatic tumors, CRC (42), and gastric cancer (43). Moreover, intracellular processes involved in cell differentiation, cell proliferation, and cancer transformation are influenced by IQGAP1 interactions with external signals (44). IQGAP1 is thought to work synergistically in cell adhesion, migration, proliferation, angiogenesis, and metastasis. IQGAP1 on the cell membrane may make adherent junctions less effective, which would encourage the dissociation and spread of tumor cells (45). The efficacy of IQGAP1 silencing using shRNA as an innovative therapeutic target for curing mouse hepatic cancer induced by DENA was investigated. This study was conducted in order to elucidate the role of IQGAP in mouse hepatic cancer progression and its relation with pro-apoptotic and antiapoptotic genes, such as the TNF-related apoptosis-inducing ligand (TRAIL) family genes, BAX, Bcl-2, caspase-3 and -9, IL-8, and CXC3, and other factors. The DENA-induced mouse liver cancer was utilized as a surrogate model for hepatic cancer. The most recent research confirms IQGAP1’s involvement in liver cancer invasion. The up-regulation of IQGAP1 in tumor cells as opposed to healthy cells has also been demonstrated (46). In light of the fact that IQGAP1 is overexpressed in tumors and consequently modulates signaling pathways involved in cell transformation, cell proliferation, and metastasis, Johnson and Henderson have proposed that IQGAP1 is a critical component of oncogenesis (44). The present data demonstrate that both the IQGAP1 protein and mRNA expression are down-regulated following treatment with IQGAP1-shRNA. Moreover, the histopathological, biochemical, and cytokine assays have shown that IQGAP1 depletion leads to a significant reduction in cell proliferation. These findings are in agreement with those of previous studies that have suggested that an IQGAP1-knockdown could attenuate cell growth (47). Our data provide direct evidence that treatment with IQGAP1-shRNA can significantly suppress the DENA-induced liver histopathological changes, increased MPO activity, increase MDA content, and inflammatory cytokine production.

The TRAIL signaling pathway is a well-established target for the treatment of liver cancer. Once the cells are stimulated by IQGAP1-shRNA, DR4 and DR5 are known to be activated and to subsequently trigger the transcription and release of pro-apoptotic genes (48). In the current study, we found that IQGAP1-shRNA can markedly attenuate the DENA-induced liver cancer development through modulation of TRAIL, IL-8 receptors, and other apoptotic factors. Growing evidence suggests that IQGAPs regulate TRAILs, IL-8, CXCR3, decoy receptors, as well as the NF-κB signaling pathway. The activation of IQGAP1 could lead to the activation of the Ras signaling pathway and the inhibition of IQGAP2, TRAIL family, and NF-κB signaling pathway. Our study has shown that the IQGAP1-knockdown regulates the expression of the aforementioned genes. The IQGAP1-knockdown modulated the liver function, interleukins, antioxidants, apoptosis, and the matrix degradation of the DENA-induced carcinogenetic process by activating both the IQGAP2 and TRAIL family genes that are capable of modulating IL-8 and its receptors.

TNF-α is known to induce the expression of matrix metalloproteinase and to increase their activity. In fact, IL-6 and TNF-α not only destroy the matrix but also inhibit the repair of the matrix (48). In our study, we found that the knockdown of IQGAP1 induces the production of pro-inflammatory cytokines that were otherwise suppressed by the DENA-induced liver cancer, through the activation of TNF-α and interleukins. Apoptosis, or programmed cell death, is a path with well-defined morphological features. The proteolytic cleavage of cellular proteins by caspases (a class of cysteine-dependent aspartate-directed proteases) results in the expression of distinctive apoptotic characteristics. Not all caspases are engaged in cell death; some have other roles to play in the body. Many physiological functions (such as tissue homeostasis) rely on naturally occurring cell death. Apoptosis dysregulation is linked to a variety of illnesses, including cancer. Apoptosis-regulating proteins, however, can be used as targets for drug development and the delivery of novel cancer treatments (47). During apoptosis, the cell survival factors Bcl-2 and Bcl-xL are also cleaved (48). Caspases are triggered during apoptosis *via* two pathways: (i) the cross-linking of death receptors in response to external (extracellular) stimuli and (ii) the release of apoptogenic substances from the mitochondria in response to internal (intracellular) signaling. The endoplasmic reticulum stress-induced apoptosis and the caspase-independent apoptosis are two further apoptotic mechanisms that exist (49). The TNF-receptor superfamily includes several plasma membrane receptors that can initiate external apoptotic signaling. In fact, the TNF-receptor-1 (TNF-R1), the death receptor-3, the TNF-related apoptosis-mediating protein, the TNF-related apoptosis-inducing ligand receptor-1 (TRAIL-R1 or DR4), the TRAIL-R2 (DR5), and the DR6 are members of this family. The binding of the receptor interaction protein, the third protein capable of interacting with the TNF-R-associated death domain protein, is known to activate the transcription factor NF-κB, thus causing antiapoptotic genes to transcribe and promote cell survival (49–52). In our study we observed that DENA increased TNF-α levels and downregulated the expression of the TRAIL receptors (DR4 and DR5) in liver cells, leading to the establishment of a higher necrosis level and, consequently, to the induction of liver cancer. However, the IQGAP1-shRNA treatment modulated this pathway by inducing the expression of DR4 and DR5, and by inhibiting TNF-α secretion. It has been proposed that the Bcl-2 protein family’s pro-apoptotic members are essential regulators of the opening of the mitochondrial permeability transition pore. The proteins of the Bcl-2 family are placed or translocated to the outer mitochondrial membrane and include both antiapoptotic (e.g., Bcl-2) and pro-apoptotic (e.g., BAX) members. The Bcl-2 family proteins are capable of regulating the release of cytochrome c by modulating the permeabilization of the inner and/or the outer mitochondrial membrane (52). The phosphorylation and the dephosphorylation of Bcl-2 family members might play an important role in the regulation of their function (53). Other proteins known to be controlled by Bcl-2 family proteins include certain caspases (namely, caspase-2, -3, and -9) as well as the apoptosis-inducing factor (AIF) (33).

Some types of cell death are difficult to categorize under “apoptosis” or “necrosis,” and there is evidence of controlled cell death with necrotic or non-apoptotic morphology. Given that apoptosis is a natural aspect of life, it is not surprising that it might be involved in the pathogenesis of a variety of disorders. Normally, apoptosis removes undesired, wounded, or virus-infected cells, but when this process is disrupted, it could result in illness. Cancer is one of the disorders linked to apoptosis suppression. Excessive cell proliferation and/or inadequate apoptosis could result in the accumulation of too many cancer cells. Defective apoptosis and malignant cell development are known to be caused by the upregulation of antiapoptotic proteins and inactivating mutations in pro-apoptotic genes (54). Several factors can activate the tumor suppressor gene p53. Many genes involved in cell cycle arrest or apoptosis (such as BAX, caspase-9, Fas, and DR5) are regulated by p53 (55). p53 has been recently shown to directly trigger the pro-apoptotic protein BAX, which in turn causes apoptosis (56).

Nowadays, most anticancer drugs in use eliminate target cells by stimulating apoptosis through receptor-mediated and/or mitochondrial-mediated mechanisms. Greater knowledge of the processes of apoptosis and apoptosis resistance has opened new possibilities for the creation of novel anticancer drugs. Most cells produce TRAIL, which interacts with cell surface death receptors DR4/5. However, because most normal cells also express decoy receptors (DcR1 and DcR2), they appear to be resistant to TRAIL-induced apoptosis (56).

Thanks to NF-κB overexpression, the NF-κB-induced expression of numerous antiapoptotic genes (such as those of Bcl-2, Bcl-xL, and c-IAP-2) has been observed in different types of cancers (57). In our model, we found that the IQGAP1-shRNA can modulate the upregulation of NF-κB induced by DENA, which is believed to have led to the initiation of the observed mouse hepatic caner. The role of IQGAP1 in HCC has been reported *in vivo* by several authors (58). Future apoptosis-based therapeutics with fewer side effects may be made possible by patient-specific profiles of genetic adaptations related to apoptosis (59, 60).

There are two opinions to investigate the importance of measuring apoptosis in nucleated blood cells as an indicator of cancer induction or suppression. The first opinion suggested that the elevation of the circulating reactive oxygen species (ROS) in blood stream which is accompanied by membrane pathology in cancer. Thus, the authors reported that the characteristic changes in cell shape of blood cells in cancer and treated-cancer cells are of value for interpreting the membrane alterations during apoptosis (61, 62). The second opinion suggested that apoptosis in peripheral blood cells was activated in 33% of breast cancer patients (63). Based on these two suggestions, we investigated apoptosis of the circulating blood cells in the cancer-induced mice to agree or disagree with this suggested phenomenon. Our results were apposite to these two old suggested theories and the apoptotic induction in all groups was not happen in the blood cells, so we cannot use the circulating blood’s apoptosis as a prognostic or a diagnostic marker in liver cancer. The basic idea behind all these interventions is the careful stimulation of apoptosis in transformed cells; an important concept that could lead to the development of new therapeutic agents that are more active and/or less toxic than the ones currently in use.

## Conclusion

IQGAP1 regulates the expression of other IQGAPs, Ras, TRAILs, IL-8 receptors, and many members of the pro- and the antiapoptotic networks. Through modulation of these genes, the silencing of IQGAP1 can improve liver function and act as an antioxidant and anti-inflammatory event and, consequently, prevent the induction of hepatic cancer by outer effectors (e.g., chemicals, radiation, etc.). Thus, the silencing of IQGAP1 could be a promising therapeutic tool against HCC.

## Data availability statement

The original contributions presented in the study are included in the article/supplementary material. Further inquiries can be directed to the corresponding author.

## Ethics statement

The animal study was reviewed and approved by National Research Centre, The Medical Research Ethics Committee has approved this study (Project number: 12060168).

## Author contributions

All authors participated in the design, interpretation of the studies and analysis of the data and review of the manuscript; KZ designed this study; AA-R, MA, AD, and KM participated in the conduct of the study. AA-R, MA, AD, KM, and KZ conducted the experiments; KZ supplied critical reagents; KZ wrote the manuscript. All authors contributed to the article and approved the submitted version.

## Funding

This study was funded by National Research Centre Supporting Project number (NRC-12060168), National Research Centre, Dokki, Egypt.

## Acknowledgments

All authors thank National Research Centre Supporting Project, National Research Centre, Dokki, Egypt.

## Conflict of interest

The authors declare that the research was conducted in the absence of any commercial or financial relationships that could be construed as a potential conflict of interest.

## Publisher’s note

All claims expressed in this article are solely those of the authors and do not necessarily represent those of their affiliated organizations, or those of the publisher, the editors and the reviewers. Any product that may be evaluated in this article, or claim that may be made by its manufacturer, is not guaranteed or endorsed by the publisher.

## References

[1] Hedman AC Smith JM Sacks DB. The biology of IQGAP proteins: beyond the cytoskeleton. EMBO Rep (2015) 16(4):427–46. doi: 10.15252/embr.201439834 PMC4388610 25722290

[2] Peng X Wang T Gao H Yue X Bian W Mei J. The interplay between IQGAP1 and small GTPases in cancer metastasis. Biomed Pharmacother (2021) 135:111243. doi: 10.1016/j.biopha.2021.111243 33434854

[3] Jameson KL Mazur PK Zehnder AM Zhang J Zarnegar B Sage J. IQGAP1 scaffold-kinase interaction blockade selectively targets RAS-MAP kinase–driven tumors. Nat Med (2013) 19(5):626–30. doi: 10.1038/nm.3165 PMC4190012 23603816

[4] Hebert JD Tian C Lamar JM Rickelt S Abbruzzese G Liu X. The scaffold protein IQGAP1 is crucial for extravasation and metastasis. Sci Rep (2020) 10(1):1–10. doi: 10.1038/s41598-020-59438-w 32051509 PMC7015931

[5] Dong PX Jia N ZJ Xu YT L DJ Li Feng YJ. Silencing of IQGAP1 by shRNA inhibits the invasion of ovarian carcinoma HO-8910PM cells in vitro. J Exp Clin Cancer Res (2008) 27(1):1–8. doi: 10.1186/1756-9966-27-77 PMC2626583 19036171

[6] Mataraza JM Briggs MW Li Z Entwistle A Ridley AJ Sacks DB. IQGAP1 promotes cell motility and invasion. J Biol Chem (2003) 278(42):41237–45. doi: 10.1074/jbc.M304838200 12900413

[7] Ren JG Li Z Sacks DB. IQGAP1 modulates activation of b-raf. Proc Natl Acad Sci (2007) 104(25):10465–9. doi: 10.1073/pnas.0611308104 PMC1965536 17563371

[8] Hu W Wang Z Zhang S Lu X Wu J Yu K. IQGAP1 promotes pancreatic cancer progression and epithelial-mesenchymal transition (EMT) through Wnt/β-catenin signaling. Sci Rep (2019) 9(1):7539. doi: 10.1038/s41598-019-44048-y 31101875 PMC6525164

[9] Liu T Zhang B Cheng X. MIB1 upregulates IQGAP1 and promotes pancreatic cancer progression by inducing ST7 degradation. Mol Oncol (2020) 15(11):3062–75. doi: 10.21203/rs.3.rs-76668/v1 PMC8564634 33793053

[10] Zhang B Cheng X Zhan S Jin X Liu T. MIB1 upregulates IQGAP1 and promotes pancreatic cancer progression by inducing ST7 degradation. Mol Oncol (2021) 21(5):1076–83. doi: 10.1002/1878-0261.12955/v2/response1 PMC8564634 33793053

[11] Al-Koussa H Atat OE Jaafar L Tashjian H El-Sibai M. The role of rho GTPases in motility and invasion of glioblastoma cells. Anal Cell Pathol (2020) 2020:9274016. doi: 10.1155/2020/9274016 PMC7013281 32089990

[12] Diao B Liu Y Zhang Y Yu J Xie J Xu GZ. IQGAP1 siRNA inhibits proliferation and metastasis of U251 and U373 glioma cell lines. Mol med Rep (2017) 15(4):2074–82. doi: 10.3892/mmr.2017.6257 PMC5365011 28259970

[13] Kawaguchi Y Fuks D Kokudo N Gayet B. Difficulty of laparoscopic liver resection: proposal for a new classification. Ann Surg (2018) 267(1):13–7. doi: 10.1097/SLA.0000000000002176 28187043

[14] Birladeanu A Rogalska M Potiri M Papadaki V Andreadou M Kontoyiannis D. Nuclear IQGAP1 promotes gastric cancer cell growth by altering the splicing of a cell-cycle regulon in co-operation with hnRNPM. bioRxiv (2020) doi: 10.1101/2020.05.11.089656

[15] Osman MA Sarkar FH Rodriguez-Boulan E. A molecular rheostat at the interface of cancer and diabetes. Biochim Biophys Acta (BBA)-Rev Cancer (2013) 1836(1):166–76. doi: 10.1016/j.bbcan.2013.04.005 PMC3667713 23639840

[16] Zhang T Wang Z Liu Y Huo Y Liu H Xu C. Plastin 1 drives metastasis of colorectal cancer through the IQGAP1/Rac1/ERK pathway. Cancer Sci (2020) 111(8):2861. doi: 10.1111/cas.14438 32350953 PMC7419044

[17] Liang Z Yang Y He Y Yang P Wang X He G. SUMOylation of IQGAP1 promotes the development of colorectal cancer. Cancer Lett (2017) 411:90–9. doi: 10.1016/j.canlet.2017.09.046 28987385

[18] Mo CF Li J Yang SX Guo HJ Liu Y Luo XY. IQGAP1 promotes anoikis resistance and metastasis through Rac1-depenDENAt ROS accumulation and activation of Src/FAK signalling in hepatocellular carcinoma. Br J Cancer (2020) 123(7):1154–63. doi: 10.1038/s41416-020-0970-z PMC7525663 32632148

[19] Bessède E Molina S Amador LA Dubus P Staedel C Chambonnier L. Deletion of IQGAP1 promotes helicobacter pylori-induced gastric dysplasia in mice and acquisition of cancer stem cell properties in vitro. Oncotarget (2016) 7(49):680–8. doi: 10.18632/oncotarget.12486 PMC5340252 27729612

[20] Lin X Kapoor A Gu Y Chow MJ Peng J Major P. Construction of a novel multigene panel potently predicting poor prognosis in patients with clear cell renal cell carcinoma. Cancers (2020) 12(11):3471. doi: 10.3390/cancers12113471 PMC7700485 33266355

[21] Wei T Choi S Buehler D Anderson RA Lambert PF. A PI3K/AKT scaffolding protein, IQ motif–containing GTPase associating protein 1 (IQGAP1), promotes head and neck carcinogenesis. Clin Cancer Res (2020) 26(1):301–11. doi: 10.1158/1078-0432.CCR-19-1063 PMC6942630 31597661

[22] Evan RD Hanna LE Junyan T Satdarshan PM Andrew WD Sayeepriyadarshini A. Scaffolding protein IQGAP1 is dispensable, but its overexpression promotes hepatocellular carcinoma via YAP1 signaling. Mol Cell Biol (2021) 41(4):e00596–20. doi: 10.1128/MCB.00596-20 PMC8088129 33526450

[23] Naidu S Shi L Magee P Middleton J Laganá A Sahooet S. PDGFR-modulated miR-23b cluster and miR-125a-5p suppress lung tumorigenesis by targeting multiple components of KRAS and NF-κB pathways. Sci Rep (2017) 7(1):15441. doi: 10.1038/s41598-017-14843-6 29133857 PMC5684387

[24] Zoheir KM Abd-Rabou AA Harisa GI Kumar A Ahmad SF Ansari MA. IQGAP1 gene silencing induces apoptosis and decreases the invasive capacity of human hepatocellular carcinoma cells. Tumour Biol (2016) 37(10):13927–39. doi: 10.1007/s13277-016-5283-8 27488117

[25] Tania N Rebecca EH Stephen AB. Quantification of mRNA using real-time RT-PCR. Nat Protoc (2006) 1:1559–82. doi: 10.1038/nprot.2006.236 17406449

[26] Seevaratnam R Patel BP Hamadeh MJ. Comparison of total protein concentration in skeletal muscle as measured by the Bradford and lowry assays. J Biochem (2009) 145(6):791. doi: 10.1093/jb/mvp037 19270056

[27] Jendrassik L. Colorimetric determination of bilirubin. Biochem (1938) 97:72–81.

[28] Doumas B Watson W Biggs H. Albumin standards and measurement of serum albumin with bromocresol green, clin. Chem Acta (1971) 31:87–92. doi: 10.1016/0009-8981(71)90365-2 5544065

[29] Beutler E Duren O Kelly BM. Improved method for the determination of blood glutathione. J Lab Clin Med (1963) 61(5):882–8. https://pubmed.ncbi.nlm.nih.gov/13967893/ 13967893

[30] Mannervik B Gutenberg C. Glutathione transferase (Human placenta). Meth Enzymol (1981) 77:231–5. doi: 10.1016/S0076-6879(81)77030-7 7329301

[31] Preuss HG Jarrel ST Scheckenbach R Lieberman S Anderson RA. Comparative effects of chromium, vanadium and gymnema sylvestre on sugar-induced blood pressure elevations in SHR. J Am Collage Nutr (1998) 17:116–23. doi: 10.1080/07315724.1998.10718736 9550454

[32] Giannopolitis N Ries SK. Superoxide dismutase. i. occurrence in higher plants. Plant Physiol (1977) 59:309–14. doi: 10.1104/pp.59.2.309 PMC542387 16659839

[33] Blagosklonny MV. Unwinding the loop of bcl-2 phosphorylation. Leukemia (2001) 15:869–74. doi: 10.1038/sj.leu.2402134 11417471

[34] Khan B Ahmad SF Bani S Kaul A Suri KA Satti NK. Augmentation and proliferation of T lymphocytes and Th-1 cytokines by withania somnifera in stressed mice. Int Immunopharmacol (2006) 6:1394–403. doi: 10.1016/j.intimp.2006.04.001 16846833

[35] Widmann C Gibson S Johnson GL. Caspase-depenDENAt cleavage of signaling proteins during apoptosis. a turn-off mechanism for anti-apoptotic signals. J Biol Chem (1998) 273:7141–7. doi: 10.1074/jbc.273.12.7141 9507028

[36] Yuan J. Evolutionary conservation of a genetic pathway of programmed cell death. J Cell Biochem (1996) 60:4–11. doi: 10.1002/(SICI)1097-4644(19960101)60:1<4::AID-JCB2>3.0.CO;2-1 8825409

[37] Balogh J Victor ID Asham EH Burroughs SG Boktour M Saharia A. Hepatocellular carcinoma: a review. J Hepatocell Carcinoma (2016) 3:41. doi: 10.2147/JHC.S61146 27785449 PMC5063561

[38] Center for Disease Control and Prevention (CDC). Hepatocellular carcinoma-united states 2001–2006. MMWR Morb Mortal Wkly Rep (2010) 59(17):517–20. 20448528

[39] Hensel J Duex J Owens C Dancik G Edwards M Frierson H. Patient mutation directed shRNA screen uncovers novel bladder tumor growth suppressors. Mol.Cancer Res (2015) 13(9):1306–15. doi: 10.1158/1541-7786.MCR-15-0130 PMC4573363 26078295

[40] Scavo LM Ertsey R Chapin CJ Allen L Kitterman JA. Apoptosis in the development of rat and human fetal lungs. Am J Respir Cell Mol Biol (1998) 18:21–31. doi: 10.1165/ajrcmb.18.1.2744 9448042

[41] Liebermann DA Hoffman B Steinman RA. Molecular controls of growth arrest and apoptosis: p53-depenDENAt and indepenDENAt pathways. Oncogene (1995) 11:199–210. https://pubmed.ncbi.nlm.nih.gov/7624128/ 7624128

[42] Shibata S Kyuwa S SK L Toyoda Y. Goto n apoptosis induced in mouse hepatitis virus-infected cells by a virus-specific CD8+ cytotoxic T-lymphocyte clone. J Virol (1994) 68:7540–5. doi: 10.1128/jvi.68.11.7540-7545.1994 PMC237198 7933139

[43] Nicholson DW Thornberry NA. Caspases: killer proteases. Trends Biochem Sci (1997) 22:299–306 12. doi: 10.1016/S0968-0004(97)01085-2 9270303

[44] Earnshaw WC LM M. Kaufmann SH mammalian caspases: structure, activation, substrates, and functions during apoptosis. Annu Rev Biochem (1999) 68:383–424. doi: 10.1146/annurev.biochem.68.1.383 10872455

[45] Martin SJ O’Brien GA Nishioka WK McGahon AJ Mahboubi A Saido TC. Proteolysis of fodrin (non-erythroid spectrin) during apoptosis. J Biol Chem (1995) 270:6425–8. doi: 10.1074/jbc.270.12.6425 7534762

[46] Hengartner MO. The biochemistry of apoptosis. Nature (2000) 407:770–6. doi: 10.1038/35037710 11048727

[47] Sakahira H Enari M Nagata S. Cleavage of CAD inhibitor in CAD activation and DNA degradation during apoptosis. Nature (1998) 391:96–9. doi: 10.1038/34214 9422513

[48] Clem RJ Cheng EH Karp CL Kirsch DG Ueno K Takahashi A. Modulation of cell death by bcl-XL through caspase interaction. Proc Natl Acad Sci USA (1998) 95:554–559. doi: 10.1073/pnas.95.2.554 9435230 PMC18458

[49] Grandgirard D Studer E Monney L Belser T Fellay I Borner C. Michel MR alphaviruses induce apoptosis in bcl-2- overexpressing cells: eviDENAce for a caspase-mediated, proteolytic inactivation of bcl-2. EMBO J (1998) 17:1268–78. doi: 10.1093/emboj/17.5.1268 PMC1170475 9482724

[50] Jin X Cai L Wang C DENAg X Yi S Lei Z. CASC2/miR-24/miR-221 modulates the TRAIL resistance of hepatocellular carcinoma cell through caspase-8/caspase-3. Cell Death Dis (2018) 9(3):1–2. doi: 10.1038/s41419-018-0350-2 29476051 PMC5833678

[51] Katrien V Dirk RVB Zwi NB. Apoptosis: mechanisms and relevance in cancer. Ann Hematol (2005) 84:627–39. doi: 10.1007/s00277-005-1065-x 16041532

[52] Whiteside ST Israel AI. Kappa b proteins: structure, function and regulation. Semin Cancer Biol (1997) 8:75–82. doi: 10.1006/scbi.1997.0058 9299585

[53] Mignotte B Vayssiere JL. Mitochondria and apoptosis. Eur J Biochem (1998) 252:1–15. doi: 10.1046/j.1432-1327.1998.2520001.x 9523706

[54] Verhagen AM Ekert PG Pakusch M Silke J Connolly LM Reid GE. IDENAtification of DIABLO, a mammalian protein that promotes apoptosis by binding to and antagonizing IAP proteins. Cell (2000) 102:43–53. doi: 10.1016/S0092-8674(00)00009-X 10929712

[55] Evan GI VousDENA KH. Proliferation, cell cycle and apoptosis in cancer. Nature (2001) 411:342–8. doi: 10.1038/35077213 11357141

[56] Sheikh MS Burns TF Huang Y Wu GS Amundson S Brooks KS. El deiry WS p53-depenDENAt and -indepenDENAt regulation of the death receptor KILLER/DR5 gene expression in response to genotoxic stress and tumor necrosis factor alpha. Cancer Res (1998) 58:1593–8. https://pubmed.ncbi.nlm.nih.gov/9563466/ 9563466

[57] Chipuk JE Green DR. Cytoplasmic p53: bax and forward. Cell Cycle (2004) 3:429–31. doi: 10.4161/cc.3.4.821 15020844

[58] Jin X Liu Y Liu J Lu W Liang Z Zhang D. The overexpression of IQGAP1 and?-catenin is associated with tumor progression in hepatocellular carcinoma in vitro and in vivo. PloS One (2015) 10(8):e0133770. doi: 10.1371/journal.pone.0133770 26252773 PMC4529304

[59] Ashkenazi A Pai RC Fong S Leung S Lawrence DA Marsters SA. Safety and antitumor activity of recombinant soluble Apo2 ligand. J Clin Invest (1999) 104:155–62. doi: 10.1172/JCI6926 PMC408479 10411544

[60] Karin M Cao Y Greten FR Li ZW. NF-kappaB in cancer: from innocent bystander to major culprit. Nat Rev Cancer (2002) 2:301–10. doi: 10.1038/nrc780 12001991

[61] Schmitt CA Lowe SW. Apoptosis is critical for drug response in vivo. Drug Resist (2001) 4:132–4. doi: 10.1054/drup.2001.0188 11512522

[62] Chukhlovin AB. Apoptosis and red blood cell echinocytosis: common features. Scanning Microsc (1996) 10(3):795–803. https://pubmed.ncbi.nlm.nih.gov/9813640/ 9813640

[63] Simão TA Andrada-Serpa MJ Mendonça GA Marques DD Braga MA Santos ALS. Detection and analysis of apoptosis in peripheral blood cells from breast cancer patients. Braz J Med Biol Res (1999) 32(4):403–6. https://pubmed.ncbi.nlm.nih.gov/10347801/ 10347801

